# Exercise-conditioned extracellular vesicles in Alzheimer’s disease: a multi-organ signaling network linking peripheral adaptation to brain pathology

**DOI:** 10.3389/fimmu.2026.1856546

**Published:** 2026-07-02

**Authors:** Rui Zhang, Kang Chen

**Affiliations:** 1 School of Humanities and Health, Bengbu Medical University, Bengbu, China; 2 Tianjin Key Laboratory of Exercise Physiology & Sports Medicine, Tianjin University of Sport, Tianjin, China

**Keywords:** Alzheimer’s disease, blood-brain barrier, exercise, extracellular vesicles, microglia, neuroimmunology, neuroinflammation

## Abstract

Alzheimer’s disease (AD) is a multifactorial neurodegenerative disorder in which amyloid-β accumulation, tau pathology, chronic neuroinflammation, cerebrovascular impairment, and synaptic dysfunction act as interconnected rather than independent processes. Physical exercise is protective against several of these features, but how its peripheral effects produce coordinated changes in the brain remains only partly defined. Soluble exerkines explain part of this benefit, but they act individually, do not protect labile cargo such as RNA, and carry little information about their cell of origin. Extracellular vesicles (EVs) offer a complementary mechanism. By packaging diverse cargo within a membrane, they co-deliver several signals at once, protect labile cargo in transit, and carry a profile that partly reflects the state and origin of the releasing cell. In this review, we develop a multi-organ signaling framework in which exercise-conditioned EVs link peripheral exercise adaptation to AD-related brain pathology. We examine how exercise reshapes EV biogenesis, the circulating EV pool, and EV engagement with the neurovascular interface. We then map how exercise-conditioned EVs intersect with amyloid aggregation and clearance, tau propagation, neuroinflammation, blood-brain barrier integrity, and synaptic and neurogenic resilience, and which tissues contribute to the exercise-responsive EV pool. Several bottlenecks keep the field at the level of association rather than causation, including cargo heterogeneity, uncertain tissue-of-origin attribution, and the gap between describing cargo and demonstrating its function. This framework outlines a realistic, staged route from current associative evidence toward clinical application, in which exercise-conditioned EVs serve first as biomarkers of exercise responsiveness and later as engineered therapeutic platforms for AD.

## Graphical Abstract

**Figure:**
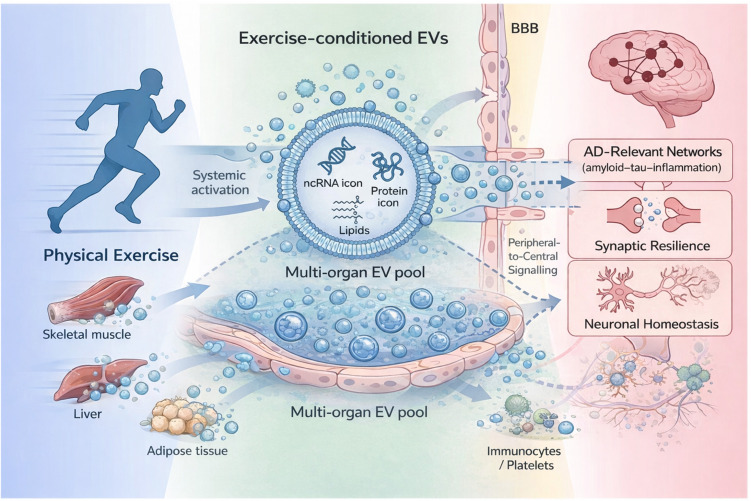

## 1 Introduction

Alzheimer’s disease (AD) is the most prevalent age-related neurodegenerative disorder and the leading cause of dementia, and its burden continues to rise as populations age (1–3). Despite decades of investigation, disease-modifying benefit has remained limited (4–6). Anti-amyloid therapies are an important advance, but their benefit has so far been modest and varies with disease stage and biological context (7–11). This has reinforced a view of AD not as a disorder of amyloid alone but as the product of several interacting processes, including tau dysregulation, chronic neuroinflammation, cerebrovascular and blood-brain barrier (BBB) dysfunction, metabolic disturbance, and synaptic failure (12–18). Because these processes are mechanistically coupled and often mutually amplifying, effective intervention is likely to depend less on any single target than on engaging the coordinated network they form.

Physical exercise is one of the most extensively studied interventions of this kind. Across clinical and experimental settings, it has been reported to benefit cognition and to act on several AD-relevant processes, including cerebral perfusion, synaptic plasticity, metabolic homeostasis, inflammatory regulation, and amyloid and tau pathology (19–27). However, how these peripheral adaptations are communicated to the brain remains incompletely defined. Most explanations have focused on soluble exerkines, namely myokines, hepatokines, adipokines, and other circulating factors released during or after exercise and carried to target tissues in the circulation (28–36). Considered individually, however, such diffusible factors are not well suited to delivering a defined combination of signals as one unit, to protecting labile cargo such as RNA, or to retaining source-associated molecular signatures. These limitations suggest that part of the systemic effect of exercise may be carried by signaling vehicles that are more structured than free soluble mediators.

Extracellular vesicles (EVs) are membrane-enclosed particles released by virtually all cell types that can transport proteins, lipids, metabolites, and nucleic acids and thereby modulate the phenotype of recipient cells, both locally and at a distance (37–40). Unlike soluble factors, EVs combine several cargo classes within a single transferable unit, conveying complex information in a coordinated rather than molecule-by-molecule manner (41, 42). Their cargo also reflects, in part, the physiological state and tissue origin of the releasing cell, providing a route through which exercise-induced peripheral remodeling could be encoded and distributed through the circulation (43, 44). Particular EV subpopulations interact with the neurovascular unit, modulate BBB function, and signal to neurons, astrocytes, and microglia (45–47). EVs have themselves been implicated in AD, where they participate in amyloid handling, tau propagation, glial activation, lipid dyshomeostasis, and vascular injury, with effects that depend on vesicle source, cargo, and disease stage (48–51). Together, these observations make EVs a plausible interface through which peripheral exercise adaptation could influence the neurovascular, glial, and neuronal processes involved in AD.

A more direct line of evidence comes from the skeletal muscle to microglia axis. In AD model mice, exercise-induced skeletal muscle-derived EVs have been reported to be taken up by microglia, where vesicle-associated miR-378a-3p contributes to amyloid plaque clearance and cognitive improvement (52). More broadly, acute exercise and chronic training reshape EV release, abundance, tissue distribution, cargo, and biological activity, producing the populations we term exercise-conditioned EVs (53, 54). Despite such links, these vesicles have rarely been considered as a single, coordinated signaling system across multiple organs in AD, a gap reinforced by their heterogeneity, uncertain tissue-source attribution, unresolved cargo-to-function causality, and limited methodological standardization (42, 54). Here, we develop this perspective into a coherent framework, from how exercise tunes EV biogenesis, through the intersections of the resulting vesicles with AD pathology, to their peripheral tissue sources. Across this arc, these vesicles emerge as a coordinated, multi-organ signaling system that links peripheral adaptation to AD brain pathology. Through this system, exercise can act on amyloid handling, tau propagation, neuroinflammation, blood-brain barrier integrity, and synaptic resilience as connected processes rather than in isolation. Framed this way, exercise-conditioned EVs become both a measurable readout of exercise responsiveness and a rational basis for engineered, brain-targeted therapeutics in AD.

## 2 Methods

Web of Science, Scopus, PubMed, and MEDLINE were searched for relevant literature published up to March 2026, including original research articles, systematic reviews, and narrative reviews, using terms such as “extracellular vesicles,” “EVs,” “exosomes,” “exercise,” “physical activity,” “exercise-induced extracellular vesicles,” “Alzheimer’s disease,” “amyloid-β,” “tau,” “neuroinflammation,” “blood-brain barrier,” “synaptic plasticity,” “neurogenesis,” and “cognitive impairment.” Eligible records were peer-reviewed primary studies and reviews addressing exercise effects on EVs, EV involvement in AD-relevant processes, or EV-mediated communication between peripheral tissues and the brain, whereas non-English articles, conference abstracts, and reports with no plausible link to neurodegeneration were excluded.

## 3 Exercise reshapes extracellular vesicle biogenesis, circulating pool dynamics, and brain access

### 3.1 Biogenesis as a tunable substrate for exercise adaptation

EV biogenesis should not be viewed as merely constitutive or passive, but as a set of partially overlapping pathways whose activity reflects the metabolic state, membrane dynamics, and stress responses of the releasing cell (37, 55–61). A major route for small EV (sEV) generation is the endosomal pathway, in which inward budding of the endosomal membrane forms intraluminal vesicles (ILVs) within multivesicular bodies (MVBs). When MVBs fuse with the plasma membrane, ILVs are released extracellularly as sEVs (56). Larger EVs (lEVs) are more commonly generated through outward budding and fission of the plasma membrane (38). This distinction between endosomal sEV formation and plasma membrane budding of lEVs is operationally useful, but it does not capture the full diversity of EV subtypes (62–66). EV composition is also not a random sample of cytosolic contents (67, 68). Cargo loading is at least partly selective and is shaped by endosomal trafficking machinery, endosomal sorting complex required for transport (ESCRT)-dependent and ESCRT-independent mechanisms, membrane lipid microdomains, and sorting regulators that enrich particular proteins, lipids, and nucleic acids in vesicles destined for release (69, 70). Because vesicle formation is coupled to selective sorting rather than indiscriminate shedding, stimuli that alter intracellular signaling may change not only the number of EVs released but also their molecular identity and signaling capacity (67, 71).

**Figure 1:**
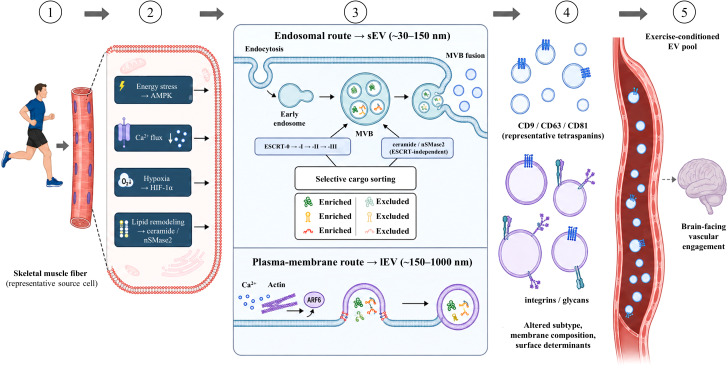
Exercise reshapes extracellular vesicle biogenesis, phenotype, and delivery potential.

Exercise is well suited to act on this machinery because it does not produce a uniform cellular response. Instead, it drives transient but recurrent shifts in substrate utilization, redox balance, calcium flux, membrane tension, and mechanical stress, all of which can influence membrane trafficking and vesicle dynamics (65, 72, 73) (**Figure 1**). Several exercise-responsive signaling axes are therefore positioned to modulate EV biogenesis (74–76). Energy stress, a defining feature of contracting skeletal muscle that can also arise in other metabolically active tissues, engages signaling centered on AMP-activated protein kinase (AMPK) (77, 78), and may affect endosomal trafficking, autophagy-related processes, and membrane turnover (79, 80). Exercise-induced calcium fluctuations extend beyond excitation-contraction coupling to influence membrane curvature, cytoskeletal reorganization, and vesicle budding, particularly in pathways linked to plasma membrane shedding (81, 82). Transient hypoxia and redox-sensitive signaling, which can develop during vigorous or sustained exercise, have been implicated in vesicle release through effects on lipid metabolism, endosomal maturation, and stress-adaptive trafficking (83–85). Ceramide-dependent mechanisms may also contribute, because exercise remodels membrane lipids and sphingolipid signaling in ways that could favor the generation of specific EV populations (85–88). These signals do not simply coexist with EV biogenesis (55, 70). They intersect with regulatory nodes that govern vesicle production and bias the molecular composition of the resulting EV pool.

Because biogenesis is itself shaped by these exercise-induced metabolic and biomechanical cues, the circulating EV pool after exercise is unlikely to be merely a scaled-up version of the resting pool. A qualitative shift is also likely (75, 89, 90), including altered subtype representation, modified membrane composition, changes in surface determinants that influence recipient-cell recognition, and biased enrichment of cargo relevant to stress adaptation, inflammation, tissue repair, and metabolic signaling (41, 91, 92). The biological significance of exercise-conditioned EVs may therefore begin upstream of cargo delivery, at the level of vesicle formation itself (89). Whether this upstream tuning translates into a durably altered circulating pool depends on how EV release and clearance are balanced across acute exercise and chronic training.

This schematic traces how exercise-associated cues (energy stress/AMPK, Ca²^+^ flux, hypoxia/HIF-1α, and lipid remodeling via ceramide/nSMase2) reshape extracellular vesicle (EV) biogenesis, cargo, and surface phenotype, using a skeletal muscle fiber as a representative exercise-responsive source cell. An endosomal route forms small EVs (sEV) as intraluminal vesicles (ILV) within multivesicular bodies (MVB), released upon MVB fusion with the plasma membrane, whereas a plasma-membrane route forms large EVs (lEV) by outward budding. Cargo is sorted selectively during biogenesis, with enriched molecules packaged into vesicles and excluded molecules retained in the cytoplasm, and representative surface markers (tetraspanins, integrins, and glycans) appear on a subset of vesicles, so the resulting exercise-conditioned EV pool differs in subtype proportion, membrane composition, and surface determinants. The dashed arrow denotes peripheral-to-central communication through neurovascular engagement rather than unrestricted blood-brain barrier (BBB) crossing. Abbreviations: EV, extracellular vesicle; sEV, small EV; lEV, large EV; ILV, intraluminal vesicle; MVB, multivesicular body; ESCRT, endosomal sorting complexes required for transport; AMPK, AMP-activated protein kinase; HIF-1α, hypoxia-inducible factor-1α; nSMase2, neutral sphingomyelinase 2; ARF6, ADP-ribosylation factor 6; BBB, blood-brain barrier.

### 3.2 Acute mobilization and chronic remodeling of the circulating pool

Exercise induces a rapid redistribution of extracellular vesicles within the circulation (93, 94), but the biological significance of this response cannot be inferred from particle counts alone. What changes is not merely EV abundance, but the organization of the circulating EV pool itself, which reflects the combined influence of tissue source, release pathway, and clearance dynamics (95–101). Acute mobilization and chronic remodeling should therefore be regarded as related but biologically distinct states, rather than as interchangeable manifestations of a single response.

The acute response is characterized by rapid kinetics. In both human and experimental settings, circulating EV levels have frequently been observed to increase during exercise or shortly after cessation, with measurable changes often detected during the early recovery period and subsequently evolving over the ensuing hours (64, 66, 102–106). The amplitude of this response is not fixed. It is shaped by characteristics of the exercise stimulus, including modality, duration, and, most consistently, intensity, with high-intensity protocols showing the clearest evidence for greater shifts in EV abundance and subpopulation profile. Such patterning is unlikely to be incidental (64, 107). Greater metabolic strain, shear stress, calcium perturbation, and membrane remodeling during more demanding exercise bouts would be expected to engage vesicle release pathways more strongly (64, 108), whereas the transient character of the response implies that mobilization is accompanied by equally dynamic processes of redistribution and clearance. The post-exercise increase in circulating EVs is therefore more appropriately interpreted as a kinetic expression of systemic adaptation than as a static feature measurable at a single time point (54).

This acute mobilization is also unlikely to reflect indiscriminate membrane leakage alone. The response is patterned rather than chaotic: it varies reproducibly with exercise intensity, differs across EV subpopulations, and can be resolved at the single-vesicle level, where increases in tetraspanin-positive vesicles have been documented after high-intensity intermittent exercise (64). Acute exercise also alters EV-associated miRNAs linked to growth, metabolism, and immune regulation, indicating that altered release is accompanied by informational bias rather than simple particulate shedding (109). Emerging human evidence further suggests that at least some exercise-responsive EV signals participate in organized inter-tissue communication, as illustrated by the transfer of muscle-associated miR-1 to adipose tissue after an acute bout of resistance exercise (106). Taken together, these observations support the view that acute exercise recruits functionally relevant EV populations through regulated deployment rather than nonspecific by-product release.

At the same time, acute EV mobilization is shaped by biological context and should not be interpreted as a uniform signature of exercise exposure. Host metabolic state appears to modify the response (110–112). In one recent study, acute aerobic exercise increased circulating exosome-like vesicles in healthy individuals under normoxic conditions, whereas this response was attenuated in prediabetic participants and under hypoxia (113). A related consideration is biological sex, as training studies have begun to reveal sex-dependent differences in subsequent acute EV responses (107). These findings suggest that exercise-induced EV dynamics are composite and state-dependent, reflecting not only the exercise stimulus itself but also the physiological landscape in which that stimulus is imposed.

Chronic training introduces a different layer of adaptation and cannot be adequately described as the repeated accumulation of acute post-exercise spikes (114). Recurrent exercise is more likely to reset the secretory phenotype of metabolically responsive tissues, thereby altering both the baseline organization of the circulating EV pool and its responsiveness to subsequent exercise bouts (115). This distinction is increasingly supported by recent evidence. Training interventions do not invariably increase resting EV abundance, yet molecular remodeling may still occur, including shifts in proteomic and miRNA profiles related to oxidative stress, inflammation, and adaptive metabolism (116, 117). The architecture of the acute EV response may also be reshaped, such that the same exercise bout elicits a different vesicular output after a period of structured training than it does in the untrained state (107). Exercise-conditioned EVs should therefore be understood not simply as vesicles sampled after physical activity, but as components of a training-shaped circulatory signaling state. Under this view, changes in particle number represent only one dimension of adaptation, while qualitative remodeling of the EV pool provides the biological basis for the phenotypic and functional questions considered in the following section.

### 3.3 Vesicle phenotype and functional delivery potential

The biological effects of exercise-conditioned EVs cannot be explained by changes in particle abundance alone. Once exercise is understood to remodel the circulating EV pool, the more consequential question becomes whether it also alters vesicle identity in ways that influence downstream signaling. Current evidence suggests that this is indeed the case. Acute exercise has been reported to shift the relative abundance of marker-defined EV subpopulations and to modify features such as vesicle size distribution and tetraspanin-associated profiles (64). These observations indicate that the post-exercise EV response is organized at the level of vesicle phenotype rather than reflecting a nonspecific increase in extracellular particles. This distinction is biologically important because EV subpopulations are not functionally equivalent (118). Differences in membrane structure and surface composition can influence vesicle persistence in circulation, bias recipient-cell uptake, and thereby shape signaling selectivity (118, 119). Phenotypic remodeling should therefore be regarded not as descriptive heterogeneity, but as a potential determinant of biological action.

The relevance of this point becomes clearer when EV delivery behavior is considered. Surface-associated properties, including tetraspanin organization, lipid composition, phosphatidylserine exposure, glycan patterning, and adhesion-related molecules, have all been linked to biodistribution and cellular uptake, even when internal cargo is similar (86, 87, 120, 121). Exercise-dependent changes in these features could therefore influence endothelial interaction, tissue retention, and recipient-cell recognition *in vivo*. At present, however, this interpretation remains more mechanistically plausible than directly established. Although exercise-induced phenotypic shifts are consistent with altered delivery behavior, direct evidence that exercise reliably drives tissue-specific targeting through defined surface remodeling is still limited. Even so, the functional relevance of phenotypic change is no longer entirely hypothetical. Human data showing EV-mediated transfer of muscle-associated miR-1 to adipose tissue after acute resistance exercise support the view that at least some exercise-responsive EV populations participate in coordinated inter-tissue communication rather than passive molecular release (106). Exercise-conditioned EVs should therefore be understood not simply as more abundant particles, but as phenotypically remodeled signaling units whose delivery properties may differ meaningfully from those of EVs at rest.

### 3.4 Peripheral-to-central trafficking and brain access

Peripheral-to-central relevance should not be equated with unrestricted passage of exercise-conditioned extracellular vesicles across the BBB (46, 122). A more informative question is whether these vesicles can engage the neurovascular interface in a regulated manner that renders central signaling biologically credible. Within such a framework, endothelial uptake, perivascular interaction, and barrier-associated signaling become relevant endpoints, even when efficient parenchymal delivery has not been demonstrated directly (46). This distinction is particularly important in the present context, because the biological significance of exercise-conditioned EVs is more likely to reside in selective neurovascular engagement than in indiscriminate access to neural tissue.

Current evidence is compatible with this interpretation. Peripheral EVs have been shown in multiple systems to interact with endothelial cells and to influence barrier-associated signaling (123, 124), suggesting that brain relevance may emerge at the level of the neurovascular unit rather than only after unequivocal translocation into neural tissue (46, 122). Recent experimental work further strengthens this view in an exercise-related setting. In hypertensive mice exposed to hypobaric hypoxia, circulating EVs derived from exercise-trained donors were more readily taken up by cerebral microvascular endothelial cells and attenuated endothelial oxidative stress and injury, indicating that exercise-conditioned vesicles can modify the cerebral vascular interface in a functionally meaningful manner (125). These findings are notable not because they prove unrestricted brain entry, but because they demonstrate that exercise-conditioned EVs can participate in regulated interactions with brain-facing vascular cells under biologically relevant conditions. This interpretation also helps distinguish exercise-associated EV signaling from conditions in which barrier dysfunction is dominated by inflammation or tissue injury. In those settings, increased permeability may permit relatively nonspecific passage of circulating material. Exercise, by contrast, is less plausibly framed as a state of generalized barrier breakdown and more plausibly understood as a context in which vesicle phenotype, endothelial responsiveness, and membrane interaction dynamics shape delivery in a selective manner (126). Under these conditions, brain access should be viewed as a graded process that may include endothelial uptake, signaling across the neurovascular interface, perivascular retention, and, in some circumstances, further propagation toward central recipient cells. What remains unresolved is not whether all exercise-conditioned EVs enter the brain efficiently, but which vesicle populations do so, by what routes, and with what degree of cell-type selectivity (46, 122).

Direct parenchymal delivery should not be treated as the sole criterion of brain relevance. Plasma-derived EVs isolated from exercised mice have been shown, when administered systemically to sedentary recipients, to enhance adult hippocampal neurogenesis (127). Although the precise trafficking route was not fully resolved, these findings indicate that peripheral exercise-derived EVs can exert brain-relevant effects at a functional level. Taken together, the available evidence supports a constrained but meaningful conclusion: exercise-conditioned EVs are biologically credible mediators of peripheral-to-central communication, even though the exact routes, efficiencies, and recipient-cell specificities through which this communication is established remain incompletely defined. This mechanistic plausibility provides the necessary foundation for examining how exercise-conditioned EVs intersect with amyloid handling, neuroinflammatory regulation, cerebrovascular integrity, and synaptic function in AD.

## 4 Mechanistic intersections between exercise-conditioned extracellular vesicles and the pathological architecture of Alzheimer’s disease

### 4.1 Modulation of Aβ metabolism

Aβ pathology is sustained not only by peptide generation but also by impaired extracellular handling and clearance (128, 129). Within this architecture, exercise-conditioned EVs act mainly at the level of clearance, modulating extracellular amyloid proteostasis rather than directly regulating amyloidogenic production. EV-associated signals can reach the machinery that governs Aβ generation, but their best-supported contribution lies downstream, in the extracellular fate of Aβ.

Several AD-relevant microRNAs illustrate how EV-associated signals can act on amyloidogenic processing. Each is dysregulated in AD, has been examined in EV-related contexts, and is mechanistically tied to amyloid generation. The miR-29 family (miR-29a and miR-29b) directly targets β-site amyloid precursor protein cleaving enzyme 1 (BACE1), the rate-limiting enzyme in Aβ generation, and engineered EV-based delivery of miR-29b reduces amyloid pathology in animal models (130–132). miR-132, which is depleted in AD brain tissue, suppresses inositol 1,4,5-trisphosphate 3-kinase B (ITPKB) signaling and thereby restrains both Aβ production and tau-related pathology (133). In a complementary approach, dendritic cell-derived EVs loaded with BACE1-targeting siRNA achieve brain-targeted BACE1 knockdown and reduce Aβ burden *in vivo (*134). Together, these studies show that EV-associated signals can engage core nodes of the amyloidogenic pathway, although they rely largely on engineered vesicles and isolated cargo rather than endogenous exercise-conditioned EVs.

More direct evidence for exercise-conditioned EV involvement in amyloid handling has emerged from animal models of amyloid burden. In an early *in vivo* study, Fuller and colleagues showed that voluntary wheel running in APP/PS1 mice improved cognition and reduced cortical Aβ42, and that intranasal administration of plasma EVs from acutely exercised wild-type donors partially reproduced exercise-associated metabolic adaptations in sedentary APP/PS1 recipients (73). The transferred EVs did not produce robust cognitive rescue, but they show that exercise-conditioned vesicles can deliver biologically active signals within an amyloid-burdened system (73). Consistent with this, blood EVs have been shown to contribute to the exercise-mediated suppression of brain Aβ pathology in App knock-in mice, reinforcing a role for circulating EVs in conveying the amyloid-related benefits of exercise (135).

Beyond carried cargo, the EV membrane itself may influence extracellular amyloid proteostasis through effects on peptide assembly. The cholesterol- and sphingomyelin-rich membrane of EVs can interfere with the fibril-elongation phase of Aβ1–42 aggregation, slowing aggregation kinetics *in vitro*. Related membrane-biophysical work indicates that Aβ assembly is sensitive to its lipid environment (136–138). EVs may therefore shape the extracellular amyloid environment not only through carried regulatory signals but also through intrinsic membrane biophysics, a route that exercise could tune by remodeling EV membrane lipids.

A further link between exercise, EV signaling, and Aβ clearance operates at the systems level of brain fluid drainage. Exercise promotes meningeal lymphatic structure and drainage capacity, enhancing bulk Aβ efflux from the brain (139, 140). In 5xFAD mice, a three-month running intervention improved meningeal lymphatic function and accelerated Aβ clearance, and this effect was partially recapitulated by serum-derived EVs from exercised donors (141). Exercise-conditioned EVs are therefore better understood as part of an integrated clearance network that spans extracellular proteostasis, vascular-fluid exchange, and lymphatic drainage, rather than as isolated anti-amyloid effectors. On balance, current evidence favors a role for exercise-conditioned EVs in shaping extracellular Aβ handling and clearance rather than in directly suppressing peptide generation, although this picture rests largely on preclinical models and still lacks interventional human confirmation.

### 4.2 Tau pathology and neuroinflammatory crosstalk

Within the AD pathological cascade, tau-related injury may be more closely aligned with exercise-associated protection than amyloid burden per se. Longitudinal positron emission tomography (PET) studies show that higher levels of physical activity attenuate Aβ-associated tau accumulation in the inferior temporal cortex, and that this attenuation mediates protection against cognitive decline more strongly than a reduction in amyloid burden does (23, 142). EV biology is central to this protection, because tau spread is increasingly understood as an intercellular process conditioned by inflammatory and metabolic states that favor pathological vesicle release and uptake (143).

Tau propagation extends beyond intracellular aggregation and depends in part on vesicle-mediated intercellular transfer, with EVs from AD brain tissue able to seed and spread tau pathology *in vivo (*143). Plaque-associated microglia have been shown to hypersecrete phosphorylated tau-enriched EVs that accelerate tau propagation in amyloid-bearing models (142). Cryo-electron tomography of human AD brain tissue further reveals that tau filaments are tethered within EV lumens rather than passively incorporated, supporting the view that EV-mediated tau dissemination is an active biological process (51). The role of lipid-regulated vesicle release is illustrated by work on the nSMase2-dependent ceramide pathway and related EV biogenesis machinery. Selective inhibition of nSMase2 in microglia prevented tau propagation in viral overexpression models, yet had limited efficacy in PS19 mice, where neuronal EV release appears to predominate (144). By contrast, a brain-penetrant, cell-type-nonselective nSMase2 inhibitor normalized tau-induced ceramide elevations, suppressed both neuronal and microglial EV release, and attenuated contralateral tau spread in the same model (145). The EV-dependent tau axis is therefore shaped not by tau pathology alone but by the inflammatory identity of the secreting cell and by the lipid-metabolic programs that regulate vesicle biogenesis and release.

Current evidence does not demonstrate that endogenous exercise-conditioned EVs directly suppress tau propagation *in vivo*. More consistently, the available data indicate that exercise acts on the permissive conditions for EV-mediated tau spread, particularly through glial activation state and sphingolipid metabolism. In non-AD inflammatory and injury models, EV-associated miR-124 can suppress TLR4/NF-κB signaling in microglia and promote more homeostatic or reparative phenotypes (146, 147), although whether exercise enriches miR-124 in AD-relevant EVs remains unknown. In 5xFAD mice, aerobic training upregulates microglial TREM2 through modulation of INPP5D, thereby suppressing cGAS-STING-mediated neuroinflammatory signaling (148). These microglial programs are not themselves EV-specific, but they shape EV-mediated tau spread, because neurodegenerative microglia are major sources of pathological vesicle secretion in tau-permissive environments (142). Exercise also acts on the lipid machinery that governs vesicle release. In a non-AD mouse model, aerobic training alters microglial ceramide synthase 1 (CerS1) expression and redirects sphingolipid flux toward C18:0 ceramide species (149), and in humans, sustained physical activity has been associated with lower circulating C18:0 ceramide levels and cognitive improvement during cardiac rehabilitation, although these data are not EV-resolved (150). Taken together, these observations place exercise within the inflammatory and lipid-metabolic programs that condition EV-dependent tau spread. The contribution of exercise-conditioned EVs in the tau axis is therefore more plausibly situated at the level of propagation competence, that is, the inflammatory and lipid-metabolic conditions that permit tau-containing EV release, uptake, and spread, rather than at the level of primary regulation of tau burden. Here too, the mechanistic steps derive from rodent and cell-culture models, whereas the human findings remain observational and do not yet identify EVs as the mediating compartment.

### 4.3 Synaptic plasticity, neurogenesis, and neuronal resilience

In AD, cognitive decline tracks more closely with the loss of synaptic and neuronal integrity than with amyloid plaque or tau tangle burden alone (151–154). Exercise-conditioned EVs are therefore most plausibly linked to functional preservation at the level of neuronal resilience. Current evidence converges on three related processes, namely preservation of synaptic plasticity, reinforcement of cell-intrinsic survival programs, and, when the hippocampal niche remains permissive, support of adult neurogenesis.

The strongest evidence currently concerns synaptic plasticity. EVs isolated from AD brain tissue carry altered synaptic proteins and pathogenic cargo that impair long-term potentiation and memory when transferred to healthy animals, indicating that vesicle-mediated signaling can directly destabilize neural circuits rather than simply reflect ongoing injury (155). Against this background, exercise-conditioned EVs shift vesicular signaling toward synaptic preservation. In a model of chronic cerebral hypoperfusion, swimming increased the release of muscle-derived small EVs enriched in miR-17/20a-5p; these vesicles crossed the BBB, accumulated in hippocampal neurons, and were associated with recovery of synaptic function (156). The effect was accompanied by suppression of DEPTOR and activation of PI3K-Akt-mTOR-related translational signaling, including downstream eIF4E phosphorylation, a pattern consistent with restoration of local protein synthesis at dendritic spines (156). Pharmacological inhibition of muscle EV release abolished both synaptic improvement and cognitive benefit, indicating that the vesicles themselves contributed to the adaptive response rather than serving only as correlates of exercise (156). Exercise also engages the FNDC5/irisin to hippocampal BDNF axis, with downstream TrkB-CREB signaling, although current evidence indicates that this axis acts mainly through soluble irisin rather than a demonstrated EV-borne route (157). Across these settings, exercise-conditioned EV signaling remains most consistently associated with preservation of synaptic adaptability in the injured brain.

Synaptic preservation alone, however, is unlikely to remain effective if neuronal viability is not maintained within the metabolically and oxidatively stressed environment of AD. A second level of relevance therefore lies in the reinforcement of intrinsic survival programs. In the ADEX randomized controlled trial, aerobic exercise increased humanin and BDNF levels in neuron-derived EVs from individuals with mild-to-moderate AD, with particularly marked responses in APOE ϵ4 carriers (24). These findings do not establish EV-specific causality, but they are biologically informative because both cargos converge on pathways central to neuronal stress resistance. Humanin has been linked to gp130/WSX-1-STAT3 signaling, which supports mitochondrial integrity and limits apoptotic activation (158), whereas BDNF activates TrkB-dependent PI3K–Akt and ERK cascades that stabilize synaptic structure and sustain activity-dependent transcriptional programs, including Arc and c-Fos (159, 160). Exercise may also alter the balance between proBDNF and mature BDNF in neural EV populations, raising the possibility that coordinated p75NTR and TrkB signaling contributes to neurite stabilization and buffering of oxidative stress under disease conditions (161). In this context, exercise-associated EV cargo is linked not only to synaptic performance, but also to the cellular programs that allow neurons to withstand persistent bioenergetic and oxidative challenge.

Adult hippocampal neurogenesis introduces a third, more conditional dimension. Exercise-derived plasma EVs have been reported to promote neural stem cell proliferation and neuronal lineage commitment in the hippocampus of young (adult) mice (127). Candidate mediators include EV-associated irisin and neurotrophic microRNAs such as miR-206 and miR-29a, which in related settings have been linked to STAT3 and PI3K signaling, induction of proneuronal regulators including NeuroD1, and enhancement of neurite outgrowth and spontaneous neuronal activity (162–164). Some of these effects appear partly independent of angiogenesis, suggesting that the neurogenic niche may be shaped directly by EV-borne signals (162–166). This regenerative axis is less uniform than either synaptic support or survival signaling. In amyloid-bearing models, EV-associated metabolic normalization, including AMPK-related signaling, has not consistently translated into meaningful neuronal rescue (41, 167, 168). Neurogenic responses therefore appear to remain constrained by inflammatory burden, niche receptivity, and disease stage, and should not be treated as an automatic downstream consequence of exercise-conditioned EV signaling across all AD-relevant contexts.

**Figure 2:**
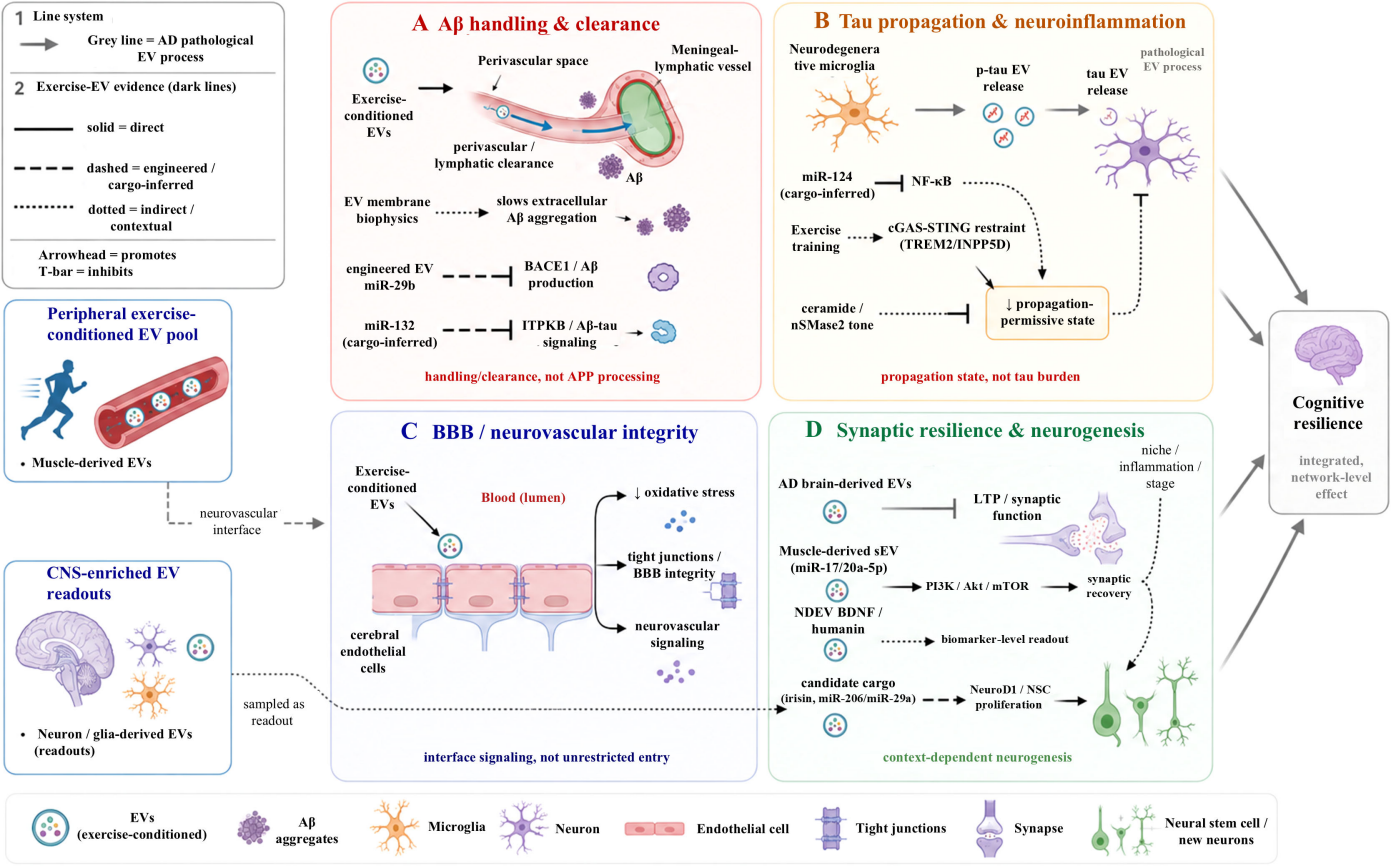
Mechanistic intersections between exercise-conditioned EVs and major pathological axes of Alzheimer's disease. This schematic summarizes how exercise-conditioned EVs intersect multiple AD-relevant pathological processes across vascular, glial, neuronal, and neurogenic compartments, engaging the brain through the neurovascular interface rather than by unrestricted blood-brain barrier (BBB) crossing. **(A)** Aβ handling and clearance: EV-associated signals act on extracellular Aβ proteostasis through perivascular and lymphatic drainage and membrane effects that may slow Aβ aggregation, rather than on APP processing. **(B)** Tau propagation and neuroinflammation: EV-mediated signaling acts on microglial activation and the propagation-permissive inflammatory and lipid state that supports vesicle-borne tau spread, rather than directly lowering tau burden. **(C)** BBB and neurovascular integrity: exercise-conditioned EVs are linked to reduced endothelial oxidative stress and preservation of tight-junction and BBB integrity, reflecting interface signaling rather than unrestricted entry. **(D)** Synaptic resilience and neurogenesis: muscle- and neuron-derived EVs support synaptic function and neuronal stress resistance through PI3K/Akt and related neurotrophic signaling, with adult hippocampal neurogenesis remaining context-dependent. Together these processes contribute to cognitive resilience as an integrated, network-level effect. Abbreviations: AD, Alzheimer's disease; EV, extracellular vesicle; sEV, small EV; NDEV, neuron-derived EV; BBB, blood-brain barrier; Aβ, amyloid-β; APP, amyloid precursor protein; BACE1, beta-site amyloid precursor protein cleaving enzyme 1; ITPKB, inositol 1,4,5-trisphosphate 3-kinase B; p-tau, phosphorylated tau; NF-κB, nuclear factor kappa B; TREM2, triggering receptor expressed on myeloid cells 2; INPP5D, inositol polyphosphate-5-phosphatase D; cGAS-STING, cyclic GMP-AMP synthase and stimulator of interferon genes; nSMase2, neutral sphingomyelinase 2; LTP, long-term potentiation; PI3K, phosphoinositide 3-kinase; Akt, protein kinase B; mTOR, mechanistic target of rapamycin; BDNF, brain-derived neurotrophic factor; NeuroD1, neuronal differentiation 1; NSC, neural stem cell; miR, microRNA.

Taken together, the available evidence is most consistent with a network-level view in which exercise-conditioned EVs support neuronal resilience across several functionally linked levels of organization (**Figure 2**). Their relevance does not lie in dominant control over a single pathogenic pathway, but in the coordination of synaptic maintenance, cellular stress resistance, and context-dependent neurogenic support within a shared adaptive framework. Exercise-conditioned EVs are therefore better understood as components of a broader intercellular signaling system that helps preserve neuronal function under progressive AD-related stress.

This schematic summarizes how exercise-conditioned EVs intersect multiple AD-relevant pathological processes across vascular, glial, neuronal, and neurogenic compartments, engaging the brain through the neurovascular interface rather than by unrestricted blood-brain barrier (BBB) crossing. At the vascular interface, EV-associated signals are linked to extracellular Aβ handling and clearance, including perivascular and lymphatic drainage and membrane effects that may slow Aβ aggregation, and to preservation of endothelial and BBB integrity, rather than to suppression of APP processing. In the glial compartment, EV-mediated signaling acts on microglial activation and the propagation-permissive inflammatory and lipid state that supports vesicle-borne tau spread, rather than directly lowering tau burden. Muscle- and neuron-derived EVs further support synaptic resilience and neuronal stress resistance through PI3K/Akt and related neurotrophic signaling, with adult hippocampal neurogenesis remaining context-dependent, collectively contributing to cognitive resilience as an integrated, network-level effect. Abbreviations: AD, Alzheimer’s disease; EV, extracellular vesicle; sEV, small EV; NDEV, neuron-derived EV; BBB, blood-brain barrier; Aβ, amyloid-β; APP, amyloid precursor protein; BACE1, beta-site amyloid precursor protein cleaving enzyme 1; ITPKB, inositol 1,4,5-trisphosphate 3-kinase B; p-tau, phosphorylated tau; NF-κB, nuclear factor kappa B; TREM2, triggering receptor expressed on myeloid cells 2; INPP5D, inositol polyphosphate-5-phosphatase D; cGAS-STING, cyclic GMP-AMP synthase and stimulator of interferon genes; nSMase2, neutral sphingomyelinase 2; LTP, long-term potentiation; PI3K, phosphoinositide 3-kinase; Akt, protein kinase B; mTOR, mechanistic target of rapamycin; BDNF, brain-derived neurotrophic factor; NeuroD1, neuronal differentiation 1; NSC, neural stem cell; miR, microRNA.

## 5 Multi-organ extracellular vesicle sources in exercise-induced neuroprotection

EV-mediated communication provides a plausible framework through which distributed exercise adaptation may be coupled to brain-relevant protection across organ systems (**Tables 1**, **2**). The key question is not whether exercise mobilizes EVs from multiple tissues, but how these tissue-defined vesicle populations differ in mechanistic status along the peripheral-to-brain axis (24, 156, 169, 216–218). Current evidence suggests that skeletal muscle is the most credible major peripheral source of exercise-responsive EVs, whereas metabolic and vascular tissues more likely shape the inflammatory, metabolic, and endothelial conditions that determine whether circulating EV signals become neurobiologically relevant. Brain-derived EVs occupy a distinct position, functioning both as local mediators of neuron–glia communication and as peripherally accessible readouts of exercise-responsive neural adaptation. The value of a multi-organ EV model therefore lies not simply in broadening the inventory of candidate exercise signals, but in explaining how tissue-specific adaptations may be integrated and rendered relevant to a disorder in which vascular, metabolic, inflammatory, and synaptic pathology are tightly coupled. At the same time, the available support remains uneven across tissue sources, and direct causal evidence for exercise-conditioned EV action is still substantially more limited than the broader body of associative and mechanistically inferred literature.

**Table 1:** Multi-organ sources of exercise-induced extracellular vesicles and their neuroprotective cargo.

Tissue origin	Characteristic protein cargo	Key miRNA cargo	Exercise response	AD/cognition-related functions	Reference
Skeletal Muscle	FNDC5/Irisin, α-sarcoglycan, HSP70	miR-1, miR-133a/b, miR-206, miR-486, miR-29a-3p	↑ Peak 10–30 min post-exercise	Induces hippocampal BDNF, enhances neurogenesis, reduces Aβ, promotes synaptic plasticity	(32, 157, 169–172)
Adipose Tissue	FABP4, leptin	miR-27a, miR-155	↑ Modality-dependent	Modulates insulin sensitivity, dysregulated AdEVs may impair cognition via metabolic inflammation	(173–175)
Liver	Fetuin-A, metabolic enzymes	miR-122-5p, miR-192, miR-22	↑ Variable, uptake site for muscle-EVs	Enhances glucose homeostasis, supports brain insulin signaling, antioxidant protection	(114, 176–181)
Vascular Endothelium	CD63, CD81, claudin-5	miR-126, miR-143, miR-145, let-7a/b/e	↑ Shear stress-induced	Preserves BBB integrity, promotes angiogenesis, reduces vascular inflammation, facilitates cargo delivery to CNS	(182–185)
Platelets	PF4 (CXCL4), tissue factor	miR-223, miR-21, miR-126	↑ Transient peak	Modulates thrombosis, inflammatory regulation	(103, 186–188)
Brain (Neurons)	Synaptotagmin, L1CAM	miR-124, miR-132, miR-9, miR-134	↑ Indirect, increased NDEV cargo after 16-week exercise in AD patients	Enhances synaptic plasticity, promotes neurogenesis, neuroprotection especially in APOE ϵ4 carriers	(24, 189–191)
Brain (Astrocytes)	GFAP, glutamate transporters	miR-26a, miR-29a, miR-181a, miR-873a-5p	↔ Context-dependent; exercise modulates phenotype	Regulates neuroinflammation, supports BBB; promotes Aβ clearance, metabolic support to neurons	(192–195)
Brain (Microglia)	TREM2, CD11b	miR-124, miR-146a, miR-155, miR-21	↓/↔ Exercise suppresses pro-inflammatory EV release	Modulates neuroinflammation, Aβ phagocytosis, synaptic pruning regulation; tau propagation control	(142, 196–198)

**Table 2:** Exercise-responsive protein and peptide cargo in extracellular vesicles with Alzheimer’s disease relevance.

Protein/peptide cargo	Functional axis	Primary EV source	Exercise response	AD-relevant target/pathway	Reference
FNDC5/Irisin	Myokine	Skeletal muscle	↑	FNDC5/irisin–BDNF signaling axis, rescues synaptic plasticity and memory deficits	(157)
Cathepsin B	Lysosomal protease/myokine	Skeletal muscle	↑	Upregulates BDNF and doublecortin (DCX) expression, promotes hippocampal neurogenesis and memory function	(199)
BDNF	Neurotrophin	Neurons (NDEVs)	↑	TrkB receptor signaling; synaptic plasticity, dendritic spine formation and circuit connectivity	(24, 200)
HSP70	Molecular chaperone	Skeletal muscle/multiple cell types	↑	Inhibits Aβ and tau misfolding and aggregation, promotes proteasomal clearance, anti-neuroinflammatory	(201)
HSP90	Molecular chaperone	Multiple cell types	↑	Tau client protein stabilization and degradation, modulates proteostasis network	(202)
αB-crystallin	Small heat shock protein (sHSP)	Skeletal muscle	↑	Inhibits Aβ fibril aggregation, anti-apoptotic, neuroprotective against amyloid toxicity	(203)
HSP27	Small heat shock protein (sHSP)	Skeletal muscle	↑	Suppresses Aβ aggregation, anti-apoptotic signaling, reduces Aβ-associated vascular damage	(204)
SOD	Antioxidant enzyme	Multiple cell types	↑	Superoxide radical dismutation, attenuates oxidative stress-induced neuronal damage, preserves neuronal population	(205)
Catalase	Antioxidant enzyme	Multiple cell types	↑	H_2_O_2_ decomposition, reduces oxidative damage, modulates serum biomarkers	(206)
GPx	Antioxidant enzyme	Multiple cell types	↑	Lipid hydroperoxide reduction, altered activity associated with vascular cognitive impairment, verbal memory biomarker	(207)
Neprilysin (NEP)	Aβ-degrading metalloprotease	Astrocytes	↑	Direct Aβ proteolytic degradation, irisin induces NEP release from astrocytes via downregulation of ERK-STAT3 signaling	(208)
Insulin-degrading enzyme (IDE)	Aβ-degrading metalloprotease	Neurons/astrocytes	↑	Aβ clearance, attenuates amyloid burden and astrocyte activation	(209)
Clusterin (apolipoprotein J)	Extracellular chaperone	Hepatocytes/plasma	↑	Complement cascade inhibition, dampens brain neuroinflammation, exercise plasma boosts memory via clusterin	(210)
Adiponectin	Adipokine	Adipose tissue	↑	Insulin sensitization, anti-inflammatory, potential mediator of pro-cognitive effects of exercise	(211)
IGF-1	Growth factor	Hepatocytes/skeletal muscle	↑	PI3K/Akt neuronal survival signaling, acute exercise increases circulating IGF-1	(212)
FGF21	Metabolic hormone/hepatokine	Hepatocytes/skeletal muscle	↑	Cerebrovascular protection, blood–brain barrier integrity, FGF21-induced neuroprotection in AD	(213)
IL-6	Pleiotropic cytokine/myokine	Skeletal muscle/immune cells	↑	Neuroinflammation modulation, IL-6 deficiency reduces neuroinflammation via inhibiting STAT3–cGAS–STING pathway	(214)
IL-10	Anti-inflammatory cytokine	Immune cells (T cells, macrophages)	↑	Microglial polarization (M2 phenotype), resolution of neuroinflammation; modulates neutrophil–microglia interactions	(215)

### 5.1 Skeletal muscle as the principal peripheral source of exercise-responsive extracellular vesicles

Skeletal muscle is currently the most credible major peripheral source of exercise-responsive EVs. Contracting muscle releases vesicles into the circulation, and a proportion of exercise-induced circulating EVs can be traced by muscle-associated markers (170, 219–221). Current support for the relevance of muscle-derived EVs to AD resides more in the convergence of candidate cargos on core disease pathways than in a fully established vesicle-specific effector mechanism (24, 135, 222, 223). Irisin and cathepsin B are representative protein cargos within this framework. Both have been detected in muscle-associated EV contexts and have independently been linked to hippocampal signaling, amyloid handling, and broader neuroprotective responses in experimental systems (95, 135, 224–229). These observations, however, do not establish that endogenous delivery through muscle-derived EVs is sufficient to reproduce such effects *in vivo*. MyomiR-enriched EV populations, including miR-1, miR-133a/b, miR-206, and miR-486, further intersect with pathways involved in amyloid precursor protein processing, neuroinflammatory regulation, and synaptic gene control (230–235) (**Table 3**). A more direct connection between skeletal muscle-derived EVs and AD-related brain effects has emerged from recent work showing that exercise-induced muscle-derived EVs can be taken up by microglia, promote plaque clearance, and improve cognition in AD models, with miR-378a-3p identified as a functionally relevant cargo (52). These data link skeletal muscle origin with brain-relevant effector activity more directly than earlier cargo-based associations. Even so, current evidence still falls short of showing that endogenous muscle-derived EVs, under physiological exercise conditions, consistently reach the brain in amounts sufficient to account for the neuroprotective effects often inferred from individual cargos. The central unresolved issue is therefore one of functional sufficiency *in vivo* rather than mechanistic plausibility in principle.

**Table 3:** Non-coding RNA cargo in exercise-responsive extracellular vesicles with relevance to Alzheimer’s disease pathology.

Type	ncRNA	Direct target(s)	Downstream effector(s)	Regulated pathway(s)	Effect	Reference
miRNA	miR-29	BACE1, NAV3, ARPC3	↓Aβ production, neuronal survival signaling	Aβ metabolism	Protective: Direct BACE1 targeting, downregulated in AD brain, exercise-restorative	(236)
miRNA	miR-34a	SIRT1, BCL2, CDK6, Tau	↓Cell survival, modulated metabolism, ↑tau phosphorylation	Apoptosis, Metabolism, Tau	Context-dependent: Elevated in AD, pro-apoptotic, exercise may modulate levels	(237)
miRNA	miR-126a-5p	VCAM-1, SPRED1, PIK3R2	↓Endothelial inflammation, ↑PI3K/Akt signaling	PI3K/Akt, NF-κB, BBB maintenance	Protective: Attenuates oxidative stress; preserves BBB integrity in AD models	(73)
miRNA	miR-129-5p	MAPT (Tau), SOX4, ITGB1	↓Tau translation, modulated neuronal differentiation	Tau pathology, Neuronal survival	Protective: Direct tau mRNA targeting, potential AD therapeutic relevance	(238, 239)
miRNA	miR-130a-3p	PTEN, ATG2B, RUNX3	↑PI3K/Akt, modulated autophagy	Autophagy, PI3K/Akt	Context-dependent: Autophagy modulation, angiogenesis	(240, 241)
miRNA	miR-132	ITPKB, p250GAP, Tau mRNA, MeCP2, SIRT1	↓BACE1, ↓GSK3β, ↑Rac1-PAK, ↑BDNF	Aβ, Tau, Synapse, Inflammation	Protective: Multi-target, most studied neuroprotective miRNA in AD, exercise-upregulated	(242)
miRNA	miR-133b	RhoA, PTBP1, MCL1	Enhanced myomiR-mediated adaptation, synaptic regulation	Synaptic plasticity, BDNF pathway	Protective: MyomiR, upregulated in acute exercise EVs, linked to cognitive benefits	(170, 243)
miRNA	miR-146a	IRAK1, TRAF6	↓Inflammatory cytokine production, ↓NF-κB activity	NF-κB, Inflammation	Protective: Reduces neuroinflammation, dual role in AD, chronic exercise modulation	(62)
miRNA	miR-148a-3p	ROCK1, DNMT1, BACE1	↓RhoA/ROCK signaling, epigenetic modulation	Aβ metabolism, Epigenetic regulation	Protective: Potential BACE1 suppression, lipid metabolism regulation	(244)
miRNA	miR-181a-5p	IGF1 signaling components	Modulated muscle adaptation, potential BDNF crosstalk	IGF1/PI3K, Inflammation	Protective (indirect): Muscle-derived, supports systemic adaptations, potential neurogenesis	(170, 245)
miRNA	miR-191-5p	DAPK1, Map3k12	↓Tau phosphorylation, ↓Aβ production, ↓MAPK signaling	DAPK1-Tau/Aβ axis, MAPK	Protective: Mitigates tau pathology and Aβ generation	(73)
miRNA	miR-192-5p	SLC39A6, p53 pathway components	Metabolic regulation, hepatic function modulation	Metabolism, Cell cycle	Protective (indirect): Liver-enriched, exercise-induced hepatic EV cargo, metabolic benefits	(179)
miRNA	miR-206	BDNF 3’UTR, HDAC4, Pax7	Modulated synaptic remodeling, ↑Neurotrophin signaling	BDNF/TrkB, Synaptic plasticity	Protective: MyomiR, promotes synaptic health via exercise EV delivery	(95)
miRNA	miR-215-5p	IDH1, BCL2L11, SIRT1	↓RIPK1/RIPK3/MLKL necroptosis pathway	Necroptosis, Oxidative stress	Protective: Inhibits neuronal necroptosis, exercise-upregulated in circulating exosomes	(246)
miRNA	miR-378a-3p	—	Microglial uptake, enhanced Aβ plaque clearance, improved cognition	Microglial phagocytosis, Aβ clearance	Protective: Muscle-derived EV cargo, taken up by microglia, promotes plaque clearance and improves cognition in AD model	(52)
miRNA	miR-451	LKB1; NLRP3	↑Metabolic stress resistance, ↓Inflammasome activation	AMPK/LKB1, NLRP3 inflammasome	Protective: Promotes neuronal survival, suppresses neuroinflammation; enriched in eEVs	(73)
miRNA	miR-532-5p	EPHA4	↓Ephrin signaling, improved tight junction integrity	BBB regulation, Ephrin signaling	Protective: Improves BBB function	(247)
miRNA	miR-3473e	Inflammatory regulators	Modulated microglial response	Inflammation	Protective: Mouse-specific, microglial regulation in exercise models	(248)
lncRNA	HOTAIR	miR-130a-3p	↓PTEN → ↑PI3K/Akt, ↓NF-κB activity, ↑ATG7, ↑LC3-II/LC3-I ratio	PI3K/Akt, Autophagy, NF-κB signaling	Protective (exercise-downregulated): ↓HOTAIR releases miR-130a-3p, restores autophagy and ↓neuroinflammation, improves MWM cognition in APP/PS1 mice	(240, 249)
lncRNA	SNHG14	miR-223-3p, miR-145-5p	↑NLRP3 inflammasome activation, ↑PLA2G4A, ↑TNF-α, IL-1β, IL-6	NLRP3 inflammasome, NF-κB neuroinflammation, Microglial M1 polarization	Protective (exercise-downregulated): Elevated in AD patients, exercise ↓SNHG14 attenuates NLRP3-mediated neuroinflammation, improves cognitive function in APP/PS1 mice	(250)
lncRNA	MALAT1	miR-382-3p	↑BDNF → ↑INSR, IRS-1, IRS-2, ↑PI3K/Akt and Ras/MAPK signaling, ↑neuronal proliferation, ↓apoptosis	BDNF/TrkB, PI3K/Akt, Ras/MAPK, Insulin signaling	Protective (exercise-upregulated in serum exosomes): Sponges miR-382-3p → ↑BDNF, rescues cognitive impairment in T2DM mice	(251)
lncRNA	NEAT1	miR-124-3p, NF-κB p65 subunit	↑BACE1 → ↑Aβ, ↑tau hyperphosphorylation, ↑pro-inflammatory cytokines (TNF-α, IL-6, IL-1β) in microglia/astrocytes	Aβ metabolism, Tau pathology, NF-κB signaling	Pathogenic (candidate for exercise-downregulation): Derepresses BACE1, promotes Aβ/tau pathology and glial NF-κB activation; knockdown improves cognition in AD models	(252)
lncRNA	BACE1-AS	BACE1 mRNA	↑BACE1 protein → ↑Aβ1-40/Aβ1–42 production, ↑amyloidogenic APP processing	Aβ metabolism (amyloidogenic pathway)	Pathogenic (candidate for exercise-downregulation): Stabilizes BACE1 mRNA, ↑Aβ production; upregulated in AD brain and plasma exosomes, potential exosomal AD biomarker	(253)
lncRNA	TUG1	miR-129-5p	↑Neuroinflammatory mediators; modulates BDNF expression, ↑hippocampal neuronal apoptosis	Neuroinflammation, Apoptosis, BDNF signaling	Protective (exercise-downregulated): Mediates exercise-induced cognitive improvement via miR-129-5p axis in APP/PS1 mice, diagnostic potential for AD	(239)
circRNA	circRIMS2	miR-186	Sponges miR-186 → ↑BDNF expression, ↓neuronal apoptosis in hippocampus	BDNF signaling, Neuronal apoptosis	Protective (exercise-upregulated): BDNF-mediated ↓neuronal apoptosis, improves cognitive performance; knockdown weakens exercise cognitive benefits (VCI model, not AD)	(254)
circRNA	circFndc3b	Enolase 1 (ENO1)	Binds ENO1 → stabilizes Klf2 mRNA (3’UTR) → ↑KLF2 protein → suppresses NLRP3 inflammasome-mediated pyroptosis, ENO1/FUS positive feedback loop promotes circFndc3b cyclization	NLRP3 inflammasome/pyroptosis, ENO1/KLF2 axis, circFndc3b/ENO1/FUS feedback loop	Protective (exercise-upregulated): Suppresses microglial NLRP3 pyroptosis via ENO1/KLF2 axis, enhances neurological recovery; silencing partially reverses exercise neuroprotection (MCAO stroke model, not AD)	(255)

↓ indicates decreased or downregulated expression/activity; ↑ indicates increased or upregulated expression/activity; → indicates a downstream or resulting effect ("leads to")

### 5.2 Metabolic and vascular tissues as complementary contributors to the circulating extracellular vesicle pool

Skeletal muscle is the best-attributed source within the exercise-responsive EV network. It does not account for the circulating pool on its own. Metabolic tissues shape that pool by influencing systemic insulin sensitivity, substrate handling, and inflammatory tone. Vascular tissues condition endothelial integrity, BBB function, and transendothelial signaling (169).

Adipose-derived EVs are closely linked to the insulin-inflammatory state that shapes AD vulnerability (256). White and brown depots release functionally distinct vesicles. White-fat EVs lean pro-inflammatory, whereas brown-fat EVs lean metabolically favorable (257). Under metabolically healthy conditions, these vesicles support systemic homeostasis and insulin responsiveness. Adiponectin has been found in them, although the evidence tying it to insulin sensitization and brain benefit comes mainly from its soluble form (112). Under obesity, adipose tissue macrophages release EVs enriched in pro-inflammatory cargo such as miR-155. These vesicles impair insulin sensitivity and amplify inflammatory signaling (174). Adipose-derived EVs can also cross the BBB. Vesicles from metabolically compromised donors aggravate hippocampal synaptic injury and cognitive impairment (175). Exercise reduces adipose tissue inflammation. This is paralleled by remodeling of the circulating EV pool (44).

The liver adds a further layer of systemic metabolic control. It governs substrate handling and endocrine-metabolic coordination at the whole-body level. Liver-derived EVs carry hepatokines, metabolic enzymes, and regulatory RNAs that couple hepatic status to distant tissues (258). Their AD relevance therefore sits at the level of systemic metabolic tone, not direct brain-directed action. FGF21 and IGF-1 are tied to exercise-responsive metabolic adaptation, and both have neurobiological effects. For these too, the supporting evidence comes mainly from their soluble forms, not their EV-resolved forms. Hepatic EVs also carry miR-122, miR-192, and related microRNAs linked to glucose and lipid metabolism (259). This role is clearest in metabolic disease. In non-alcoholic fatty liver disease, circulating EV profiles shift toward pro-inflammatory, insulin-resistant signatures (260). This condition is also linked to neuroinflammation and cognitive impairment through chronic systemic inflammation (261). Exercise partly reverses this trajectory. It improves hepatic metabolic function and remodels liver-associated EV profiles (44).

Brain relevance is further conditioned by the vascular interface. Through it, peripheral EV signals meet the central nervous system. Endothelial-derived EVs reflect and influence BBB integrity, angiogenic balance, and vascular homeostasis (123). Their cargo includes miR-126 and the miR-143/145 cluster. Peripheral EV signaling thus depends not only on what enters the circulation. It also depends on the state of the endothelium and BBB that receive it (123). Exercise-associated remodeling of endothelial EVs is most plausibly linked to preserved BBB integrity, vascular stability, and regulated neurovascular communication (187). Platelet-derived EVs influence thrombosis, endothelial interaction, and inflammatory tone. Immune cell-derived EVs track shifts in inflammatory activation and modify vascular and tissue signaling more broadly (187). Current evidence places both within the hemostatic and inflammatory environment that carries other peripheral EV signals, not among the major exercise-induced neuroprotective effectors.

Metabolic and vascular tissues therefore do not act as equivalents of skeletal muscle. Adipose tissue and liver set the systemic conditions that favor or oppose insulin sensitivity, metabolic flexibility, and inflammatory restraint. Vascular tissues decide whether those signals meet a brain-facing interface able to keep barrier integrity and neurovascular balance. Their contribution to AD is expressed mainly through the conditions under which peripheral EV signaling gains, or fails to gain, brain relevance.

### 5.3 CNS-derived EVs as intrinsic mediators and biomarker readouts

CNS-derived EVs operate within the disease-relevant communication network of the AD brain (262–264). They occupy a dual role, acting locally as carriers of neuron-glia signals and persisting in the circulation as accessible markers of those neural states (244, 265–268).

Neuron-derived EVs carry synaptic proteins, neurotrophic factors, and regulatory microRNAs that support neuronal maintenance, circuit communication, and plasticity (189, 269, 270). In individuals with AD, exercise-induced changes in neuron-derived EV cargo have been detected after training (24), suggesting that selected adaptive neuronal programs remain inducible even in the degenerating brain.

Whether that signal is sustained depends on the surrounding glia. The metabolic and inflammatory context of the CNS is shaped in part by astrocyte- and microglia-derived EVs (198, 271, 272). Astrocyte-derived EVs deliver antioxidant enzymes, metabolic substrates, and protective RNAs, whereas microglial EVs either amplify inflammatory injury or promote debris clearance depending on their activation state (263). Exercise is therefore unlikely to act on neuronal EVs alone, and more likely remodels neuron- and glia-derived populations together, with combined consequences for synaptic stability and inflammatory restraint (73, 273, 274).

For now, the peripheral accessibility of these vesicles is more useful diagnostically than mechanistically. Available evidence supports their use as readouts of otherwise inaccessible CNS states rather than as a major route of brain-to-periphery effector traffic (267, 268). In AD, where brain tissue cannot be sampled repeatedly, such readouts offer a practical way to track whether exercise shifts molecular programs toward resilience rather than decline.

## 6 From association to causation: unresolved challenges

What currently limits the field is not a shortage of exercise-responsive EV observations, but the absence of experimental architectures capable of linking source, cargo, potency, and biological effect within the same causal model. Much of the literature already supports the view that exercise reshapes EV release, composition, and disease-relevant signaling (101, 169, 275–277). The unresolved issue is whether those observations can be assigned to defined vesicle populations, traced to specific tissue origins, connected to functionally decisive cargo constellations, and reproduced as measurable neuroprotective effects in AD-relevant systems. Until these levels of evidence are integrated, mechanistic interpretation will remain stronger at the level of plausibility than at the level of causal resolutions.

A first constraint arises at the level of vesicle identity. Exercise mobilizes EVs from multiple tissues simultaneously, generating a circulating pool that is heterogeneous in origin, composition, and temporal dynamics. This heterogeneity is not merely an analytical inconvenience; it directly limits causal inference. Even when an exercise-associated EV phenotype is reproducibly detected, attribution of that phenotype to a specific tissue source often remains probabilistic rather than definitive (42, 55, 187, 278, 279). Surface-marker enrichment improves resolution but does not confer absolute tissue specificity, and bulk isolation collapses functionally distinct subpopulations into composite samples. These limitations are especially consequential in a field that aims to explain how peripheral exercise adaptation becomes relevant to brain protection. Vesicles enriched for muscle-associated markers may still contain endothelial, immune, platelet, hepatic, or adipose contributions, particularly after acute exercise when release is widespread and rapidly changing. Similar caution applies to blood-derived CNS-enriched EV fractions, where marker-based capture increases interpretability without eliminating overlap, incomplete recovery, or contamination (42, 55). Source heterogeneity therefore limits mechanistic inference at a structural level. Claims regarding tissue of origin and organ-specific EV function will remain provisional until source-resolved tracing and subpopulation-level analysis become more routine.

This problem persists even after a vesicle population has been linked to a plausible source. Cargo-level interpretation is frequently more precise than the available evidence can justify. Functional effects are often assigned to a single microRNA, protein, or peptide, yet native EVs carry combinatorial repertoires whose biological activity is likely to depend on stoichiometry, co-packaging, delivery context, and interactions among multiple molecular species (280, 281). Demonstrating that one cargo increases after exercise, and that the same molecule has neuroprotective activity in another setting, does not establish necessity or sufficiency within the vesicular context itself. In many cases, the field has identified molecules with plausible relevance to AD before establishing whether those molecules are functionally decisive in the native EV population from which they are inferred. This gap is particularly evident in mechanistic animal studies that rely on ex vivo conditioned vesicles, engineered vesicles, or cargo-focused manipulations as substitutes for bona fide exercise-generated EVs *in vivo*. Such approaches are often informative, but they shift the evidentiary balance toward association and pathway accessibility rather than EV-specific causality. The key challenge is therefore not simply to identify candidate cargos, but to determine which cargo constellations are necessary, which are sufficient, and which are only correlative features of broader exercise-responsive vesicle states.

Once the problem is framed at the level of native vesicle biology, the next unresolved layer is functional potency. Conventional EV characterization metrics such as particle yield, size distribution, or marker positivity do not adequately define biological activity. Single-analyte assays are likewise poorly suited to vesicle preparations whose effects emerge from coordinated signaling across multiple cargo classes. A preparation can therefore be analytically reproducible without being biologically equivalent. This limitation becomes especially important for translational efforts, including engineered or exercise-mimetic EV platforms, where reproducible identity and purity do not guarantee reproducible neuroprotective efficacy. What remains missing are potency frameworks that relate defined vesicle features to reproducible outputs in AD-relevant systems, including effects on synaptic maintenance, neuroinflammatory regulation, cerebrovascular stability, metabolic resilience, and disease-relevant behavior. Without such frameworks, standardization will remain technically rigorous but biologically incomplete.

These uncertainties converge at the level of study design. Additional descriptive cataloging is unlikely to resolve them. Progress depends on experimental designs that align exercise paradigms, EV isolation strategy, source-resolved tracing, gain- and loss-of-function testing, subpopulation-level analysis, and potency measurement within the same biological question. Such studies are still uncommon. Much of the current literature remains organized around exercise-associated cargo shifts or inferred relevance based on overlap with known protective pathways, whereas mechanistically aligned intervention studies remain limited. What is needed are designs in which exercise-conditioned or engineered EVs are tested as independent variables, with prespecified source characterization, cargo definition, dose control, and outcome measures matched to a clear mechanistic hypothesis. In preclinical work, this will require efficacy testing across models that capture the complexity of AD, including amyloid and tau pathology, neurovascular dysfunction, aging, and metabolic comorbidity. In clinical translation, it will require biomarker-stratified and longitudinal frameworks capable of identifying which patient subgroups show measurable vesicle responsiveness and which EV signatures track with meaningful biological or clinical benefit. Regulatory progress will likewise remain slow until identity, potency, and reproducibility can be defined with sufficient precision to support platform-level comparability.

The central methodological problem in the field is therefore not insufficient descriptive richness, but insufficient causal integration. Exercise-responsive EV biology is already conceptually relevant to AD. What remains unresolved is which vesicle populations are mechanistically decisive, which molecular ensembles confer biological activity, and under what experimental conditions those properties can be translated into reproducible neuroprotective effects. Resolving these questions will determine whether the field advances beyond plausible association and toward a genuinely intervention-ready framework.

## 7 Toward translation: biomarkers, therapeutics, and the road ahead

**Figure 3:**
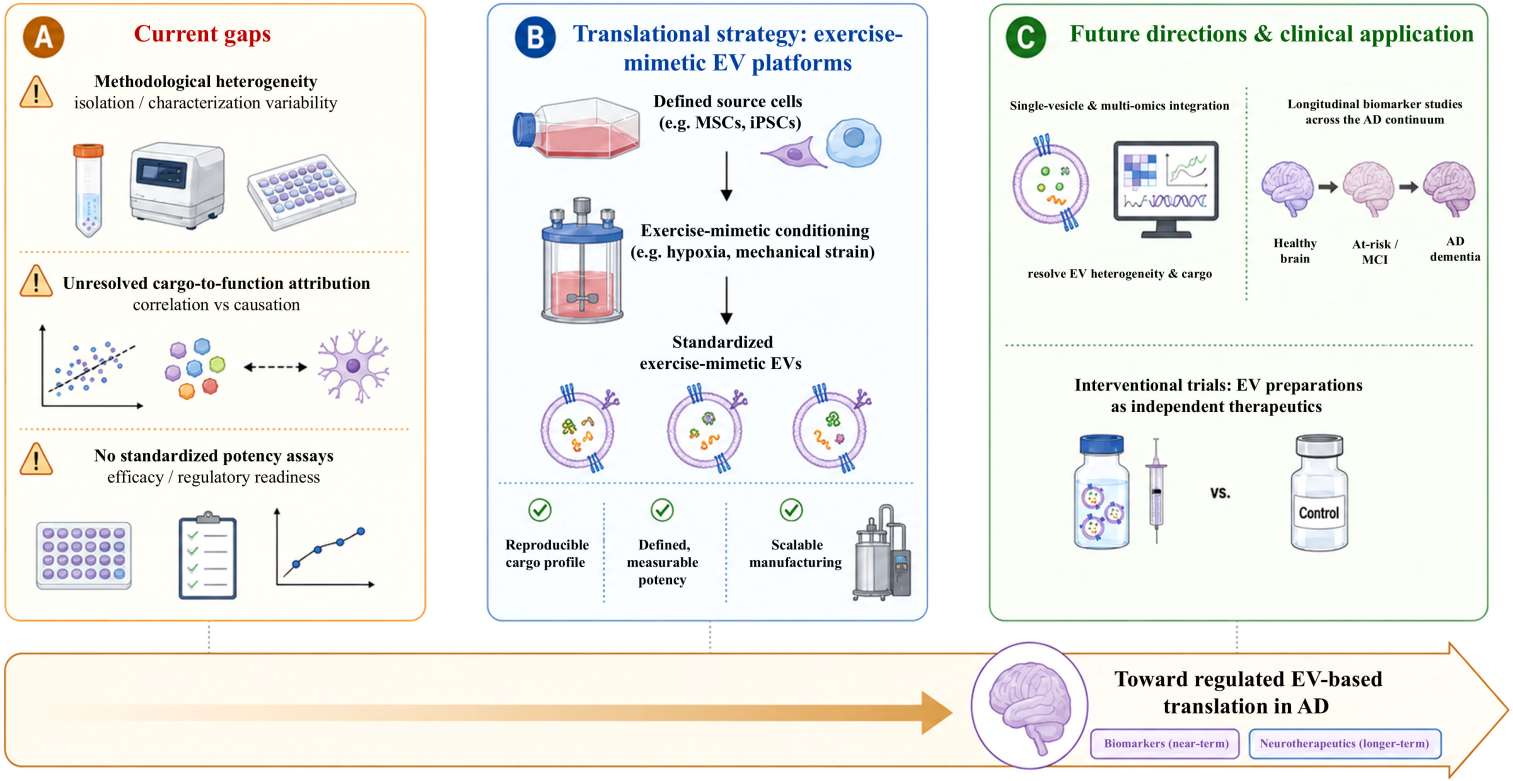
Translational prospects of exercise-conditioned extracellular vesicles in Alzheimer’s disease. This schematic outlines the main barriers and future directions for translating exercise-conditioned extracellular vesicle (EV) biology into AD applications, and highlights the field’s asymmetry, in which biomarker use is nearer-term and therapeutic use is longer-term. **(A)** Current gaps include methodological heterogeneity in EV isolation and characterization, unresolved attribution of cargo to function, and the absence of standardized potency assays. **(B)** A plausible therapeutic route is the development of exercise-mimetic EV platforms produced from defined source cells under controlled conditioning, with reproducible cargo composition, defined and measurable potency, and scalable manufacturing. **(C)** Progress will depend on resolving EV heterogeneity through single-vesicle and multi-omics approaches, embedding EV profiling in longitudinal biomarker studies across the AD continuum, and testing EV preparations as independent candidates in interventional trials, together supporting the development of regulated, mechanism-guided EV-based neurotherapeutics. AD, Alzheimer’s disease; EV, extracellular vesicle; MSC, mesenchymal stromal cell; iPSC, induced pluripotent stem cell; MCI, mild cognitive impairment.

The translational future of exercise-conditioned EVs in AD is inherently asymmetric. Their value is already evident as minimally invasive readouts of biological responsiveness to exercise, whereas therapeutic development remains limited by unresolved challenges in vesicle definition, cargo control, delivery reproducibility, and functional potency (**Figure 3**). Biomarker application is therefore the more immediate translational path. Exercise-responsive changes in circulating EVs, particularly within neuron- and glia-enriched fractions, offer a tractable window into otherwise inaccessible CNS adaptation and may serve as indicators of target engagement and intervention responsiveness in longitudinal studies. By contrast, therapeutic translation is more demanding because native exercise-derived EVs are biologically informative but poorly suited to direct clinical development as standardized products. A more plausible route is the development of function-defined exercise-mimetic vesicle platforms with controllable composition, reliable brain-directed activity, and potency measures linked to AD-relevant outcomes. In this respect, EVs are likely to mature first as biomarkers of exercise biology and only later as clinically reliable therapeutic agents.

## 8 Conclusions

On current evidence, exercise-conditioned EVs are best viewed as part of an emerging coordinated, multi-organ signaling framework that links peripheral exercise adaptation to AD-related brain pathology. Exercise acts on EV biology at several levels, tuning vesicle biogenesis, remodeling the circulating pool across acute and chronic timescales, and altering vesicle cargo and surface phenotype. It also shapes vesicle engagement with brain-facing vascular and barrier compartments. In AD-relevant settings, these vesicles are most consistently linked to extracellular amyloid handling and clearance, propagation-permissive inflammatory and lipid-metabolic states in the tau axis, endothelial and blood-brain barrier signaling, and synaptic resilience. Neurogenic effects appear more context dependent. These effects should not be attributed to a single cargo or a linear pathway. Their shared feature is coordinated, vesicle-borne communication across peripheral and central compartments.

This communication does not arise from one tissue. Skeletal muscle currently provides the strongest direct peripheral evidence, whereas adipose tissue, liver, vascular compartments, platelets, and immune cells appear to shape the metabolic, inflammatory, and endothelial milieu in which EV signals acquire brain relevance. CNS-derived, or CNS-enriched, EV populations occupy a distinct position. Within the brain, they participate in neuron-glia communication. In the circulation, they serve as accessible readouts of exercise-responsive neural states. The net effect on the brain is therefore likely to reflect the combined output of several tissue sources rather than the action of a single dominant organ.

The strength of this evidence remains uneven. Much of it is associative or preclinical, or based on engineered and cargo-focused models rather than on endogenous vesicles generated by physiological exercise. Direct human evidence is currently strongest at the biomarker level, with target engagement still largely inferred. Causal evidence for disease modification remains largely preclinical. Source attribution, cargo causality, brain-facing delivery routes, and functional potency are still incompletely resolved. Progress will require source-resolved tracing, subpopulation-level analysis, gain- and loss-of-function testing, and potency measures matched to AD-relevant outcomes.

Biomarker development is therefore the most immediate translational opportunity. Exercise-responsive changes in circulating EVs, particularly in neuron- and glia-enriched fractions, may offer a practical window into CNS adaptation and intervention responsiveness. Therapeutic use is a longer-term prospect and will depend on function-defined, exercise-mimetic or engineered EV platforms with controlled composition, reproducible delivery, and validated potency. The implications also extend to aging. Chronic neuroinflammation, cerebrovascular dysfunction, impaired proteostasis, synaptic failure, and declining intercellular communication are shared features of the aging brain. By potentially helping to preserve or re-engage structured intercellular communication, exercise-conditioned EVs may contribute to resilience in the aging brain beyond AD-specific pathology. Defining that contribution will also set the evidentiary standards needed to translate this biology into biomarkers and future interventions.
